# Cortical Bone Trajectory Screws in Posterior Lumbar Interbody Fusion: Minimally Invasive Surgery for Maximal Muscle Sparing—A Prospective Comparative Study with the Traditional Open Technique

**DOI:** 10.1155/2018/7424568

**Published:** 2018-02-18

**Authors:** Nicola Marengo, Marco Ajello, Michele Federico Pecoraro, Giulia Pilloni, Giovanni Vercelli, Fabio Cofano, Francesco Zenga, Alessandro Ducati, Diego Garbossa

**Affiliations:** Department of Neuroscience, Neurosurgery Section, University of Turin, Turin, Italy

## Abstract

**Introduction:**

A prospective comparative study between classical posterior interbody fusion with peduncular screws and the new technique with divergent cortical screws was conducted.

**Material and Methods:**

Only patients with monosegmental degenerative disease were recruited into this study. We analyzed a cohort of 40 patients treated from January 2015 to March 2016 divided into 2 groups (20 patients went to traditional open surgery and 20 patients under mini-invasive strategy). Primary endpoints of this study are fusion rate and muscular damage; secondary endpoints analyzed were three different clinical scores (ODI, VAS, and EQ) and the morbidity rate of both techniques.

**Results:**

There was no significant difference in fusion rate between the two techniques. In addition, a significant difference in muscular damage was found according to the MRI evaluation. Clinical outcomes, based on pain intensity, Oswestry Disability Index status, and Euroquality-5D score, were found to be also statistically different, even one year after surgery. This study also demonstrated a correlation between patients' muscular damage and their clinical outcome.

**Conclusions:**

Cortical bone trajectory screws would provide similar outcomes compared to pedicle screws in posterior lumbar interbody fusion at one year after surgery, and this technique represents a reasonable alternative to pedicle screws.

## 1. Introduction

**Figure 1:**
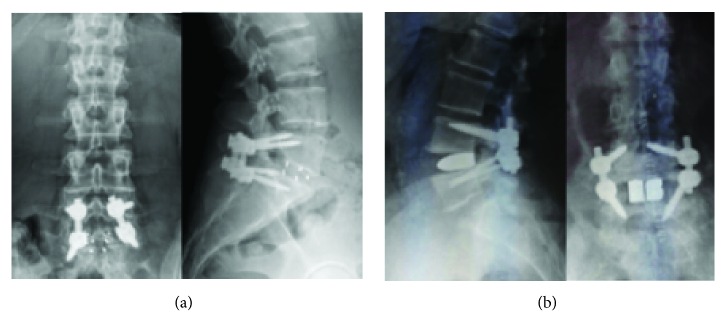
Postoperative radiograms showing anteroposterior and lateral view after cortical bone trajectory-PLIF (a) and after pedicle screws-PLIF (b).

We performed a prospective comparative study with one-year minimum follow-up to determine if cortical bone trajectory (CBT) screws are equivalent to even better posterior stabilizers in PLIF surgery compared to pedicle screws (PS) on the basis of clinical and radiological outcomes (Figure 1).

Primary study endpoints were fusion rate and muscular damage. There were several secondary endpoints: they included the maximum intensity of back/leg pain (measured using the visual analogue scale), functional status (using the Oswestry Disability Index and the Euroquality-5D score), surgical morbidity (based on operative time, estimated blood loss, radiation exposure, length of stay, and early complications such as screw malpositioning, infection, dural tear, superior facet joint violation, and new neurological deficit), and additional outcomes including mechanical failure (screws pullout and/or cage subsidence). All these data were collected prospectively both preoperatively and at each follow-up visit by the same surgeon.

## 2. Materials and Methods

Twenty patients were included in the CBT group and in the traditional pedicle screw group each of whom fully complied with the inclusion criteria.

Inclusion criteria were as follows: degenerative monosegmental lumbar disease (lumbar stenosis with severe foraminal stenosis, herniation recurrence with discopathy, first grade degenerative spondylolisthesis according to Meyerding classification system) by using computed tomography scans, magnetic resonance, and standing and functional radiographs. Patients were required to have shown no improvement in clinical symptoms despite several conservative treatments (including medication, physical therapy, and injection treatment) over a period of 6 months or more. Patients were required to have undergone posterior lumbar interbody fusion at a single level with posterior fixation with screws (cortical bone trajectory or traditional pedicle screws) and interbody polyetheretherketone (PEEK) or porous tantalum cages. Patients were aged between 30 and 70 years and finally they were required to complete a year or longer follow-up period. Fractures, infections, or tumors in the lumbar spine even if at a different motion segment, osteoporosis diagnosed by a *T* score less than −2,5 on dual-energy X-ray absorptiometry bone densitometry measurements, multilevel fusion surgery, hemorrhagic disorders such as hemophilia and thrombocythemia, chronic daily steroid therapy (more than one year), inability to accurately complete preoperative and postoperative questionnaires and lack of completion of all radiological assessment examination at one year after surgery (CT scan, MRI, and static and dynamic radiograms of the lumbar spine) represented exclusion criteria.

As we started on April 2015, at the end of 2016, 20 patients with CBT-PLIF completed one-year follow-up period; at the same time 23 patients with PS-PLIF completed the follow-up. As this study is not randomized the surgical indication depended on the surgeon and was not influenced by the patient's will in any case. Naturally, both the physician and the patients were aware of which kind of operation they were undergoing. Patients were found to be similar between groups with respect to demographic characteristics such as age, gender, smoking status, body mass index (BMI), preoperative lumbar pathology, bone minerality index (*T* score), duration of symptoms, and distribution of surgical levels treated. Four cases from the CBT and three of the PS group were fused using PEEK cages instead of tantalum cages but this was not statistically significant (Table 1).

Every patient was studied with preoperative flexion-extension standing X-ray to evaluate macroscopic segmental spinal instability. We found macroscopic spinal instability in two patients affected by isthmic spondylolisthesis (10% in PS-PLIF group, *p* = 0.4872) [1, 2].

We preferred using pedicle screws in patients with spondylolisthesis because we had no previous experience on this pathology using CBT technique.

All procedures were performed by a single neurosurgeon who used the same operative technique for each surgery. Posterior decompression via partial laminectomy was performed through a posterior midline incision and screws were positioned according to the different techniques. When necessary a partial or total medial facetectomy was also performed.

For S1 vertebra, the entry point was located 3 mm caudal to the most inferior border of the descending L5 articular process, cranially angulated towards the anterosuperior sacral edge. Thus our technique differs from the one described by Matsukawa et al. because there is no violation of sacral endplate [3].

In each patient two cages were routinely used with the autograft bone materials that were locally obtained during posterior decompression. In the CBT group in 16 cases porous tantalum cages were used (Ardis Zimmer, Warsaw, IN, USA) and in 4 cases PEEK cages (MAS system, Nuvasive, San Diego, CA, USA); in the PS group only 3 cases had PEEK cages (Concorde, Synthes, West Chester, PA, USA) while for 17 patients tantalum cages were used (Ardis Zimmer, Warsaw, IN, USA). In the CBT group bilateral screw-rod system with cortical bone trajectory screws (MAS System Nuvasive, San Diego CA, USA) was used under fluoroscopic guidance and in two cases with the help of a neuromonitoring system (NVM5 Nuvasive, San Diego, CA, USA); 5.5 × 30 or 5.5 × 35 mm screws were used for all levels treated. In the other group, bilateral screw-rod system with pedicle screws (Expedium, DePuy, Warsaw, IN, USA) was used under fluoroscopic guidance; 6 × 40 or 6 × 45 mm screws were used or all levels treated.

Patients in both groups were admitted to the same wards after surgeries and were treated with the same postoperative protocols. All patients were permitted to ambulate the first day after surgery in some cases wearing a soft corset according to the patients' will. Most of the patients in both groups were discharged from the hospital by 7th postoperative day and were encouraged to avoid sitting for more than one hour a day during the first month after surgery and after 3 months they were allowed to start back normal daily activities, including heavy lifting.

All the data regarding the surgery and the hospital stay were collected including average blood loss, length of stay, surgery duration, total radiation dose area product, and early complications (screw/cage malpositioning, infection, dural tear, root damaging, etc.). Moreover the patients were required to complete three main questionnaire (Visual Analogue Scale, Oswestry Disability Index, Euro Quality 5D) preoperatively and at the dismission. Standing lumbar radiograms in flexion and extension are obtained before the discharge and at 3, 6, and 12 months after surgery. A follow-up CT scan is suggested at 12 months to assess fusion. Moreover we suggested a lumbar MRI at 12 months to assess muscular damage. At the same time they were asked to complete the questionnaires.

Fusion status was determined only at 12 months after surgery. The treated segment was considered fused if the difference between the Cobb angles in lateral radiographs in flexion and extension views was less than 2° or at least a bony bridge was evident at the CT scan. Nonunion was declared whether the difference in Cobb angle was greater than 2° or if there was not any bony bridge on CT scans. A neuroradiologist, who was not involved in patients' treatment, performed all dynamic radiograms and CT measurements and reconstructions. All CT scans were changed to the bone window level to better recognize true bony bridges between endplates and were taken using a 1 mm interval.

Muscular damage at the operative level was determined according to a T2-weighted MRI images; MRI was performed on al least a 1.5 Tesla MRI system preoperatively and at the final follow-up, 1 year postoperatively. All images were obtained using a T2-weighted fast spin echo sequence. Slice thickness ranged from 4 to 6 mms and interslice gap was 1 mm. We used anatomic markers and locating lines on sagittal plane scans to select the most similar preoperative and follow-up axial images, at the same spinal level, for comparison. To determine the lean multifidus muscle cross-sectional area (CSA), the region of interest (ROI) was drawn around the multifidus muscles bilaterally, taking care to avoid nearby fat, bony structures, and other soft tissues. The sum of CSAs of bilateral lean multifidus was calculated. To determine the mean signal intensity of multifidus, the ROI was drawn around the outer perimeter of the muscle unilaterally, to include any areas of intramuscular fat. Mean signal intensity of unilateral gross multifidus muscle on a T2-weighted axial image was evaluated quantitatively by the grayscale histogram plugin of Osirix, in which a higher score means higher signal intensity. The mean signal intensity of psoas muscle in the same axial image was also evaluated as control from a 100 mm2 circular ROI placed in the center of the muscle. The signal intensity ratio of gross multifidus to psoas was calculated. After we obtained the multifidus CSA and the T2 ratio preoperatively and at the final follow-up we calculated the percentage changes for both kinds of datasets. Apart from straight comparison between radiological/clinical outcomes and surgical morbidity we hypothesized a possible correlation between the amount of muscular damage and clinical outcomes. We supposed that these two variables were correlated as follows: worse outcomes were related to greater muscular damage.

Independent Student's *t*-tests were used for continuous variables and the Fisher exact test was used for proportional variables. Two-sided *p* values < 0.05 were considered to be statistically significant. The supposed correlation between paraspinal muscular damage and clinical outcomes was analyzed calculating Pearson's index. All statistical analyses were performed using Apple Numbers and Microsoft Excel software.

## 3. Results

According to the dynamic radiographs and to the lumbar CT scans evaluated at 12 months after surgery, fusion was achieved in 18 of 20 patients (90%) in the CBT group and in 17 of 20 patients (85%) in the PS group. The difference in fusion rate was not significant (*p* = 0,3292) (Table 2). According to lumbar MRI performed preoperatively and at 12 months after surgery we evaluated the muscular damage with two different parameters: the multifidus cross-sectional area (MF-CSA) that represents the true, functional, and fatty-free area of the multifidus muscle on T2 axial image and the operating level and the T2 MF-psoas ratio that represents the ratio between the mean T2 signal of MF area and 1 cm2 in the center of the psoas muscle.

The CSA of lean multifidus muscle at the operative and adjacent levels had decreased at final follow-up in both CBT and PS groups. The percentage changes were greater in the PS group at the operative level (16,76% versus 24,59%, *p* = 0.0483). When further analyses were run separately for the men and women in each group, the differences between groups were still evident. The signal intensity ratio of gross multifidus to psoas had increased at the operative levels in both groups at final follow-up. The percentage change in the ratio at 12 mo FU was larger in the PS group (9.35% versus 23.96%, *p* = 0.0028). When further analyses were run separately for the men and women in each group, the differences remained statistically significant.

**Figure 2:**
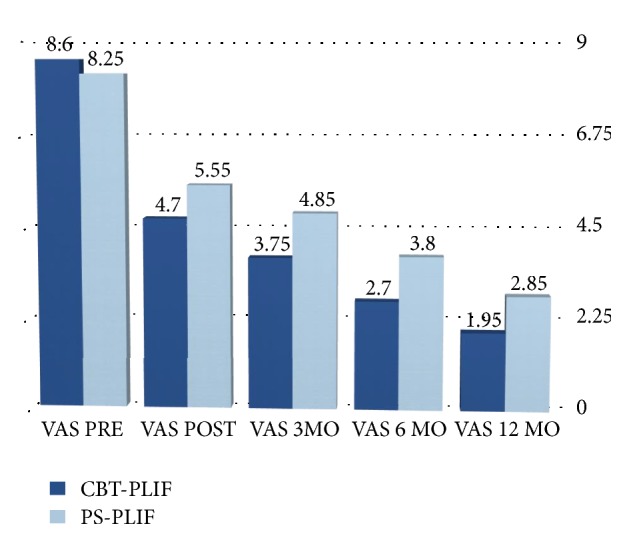
VAS scores on admission and at the discharge.

Mean VAS score for low back pain indicated that pain levels at the discharge were significantly lower than preoperative ones for both groups, with mean scores decreasing from 8.6 (SD 1.19) to 4.7 (SD 2.08) in CBT group and from 8.25 (SD 1.27) to 5.55 (SD 1.50) in PS group with a *p* value < 0.001. The VAS scores at the discharge were also found to be significantly different between the two groups (*p* = 0.0471) (Figure 2).

**Figure 3:**
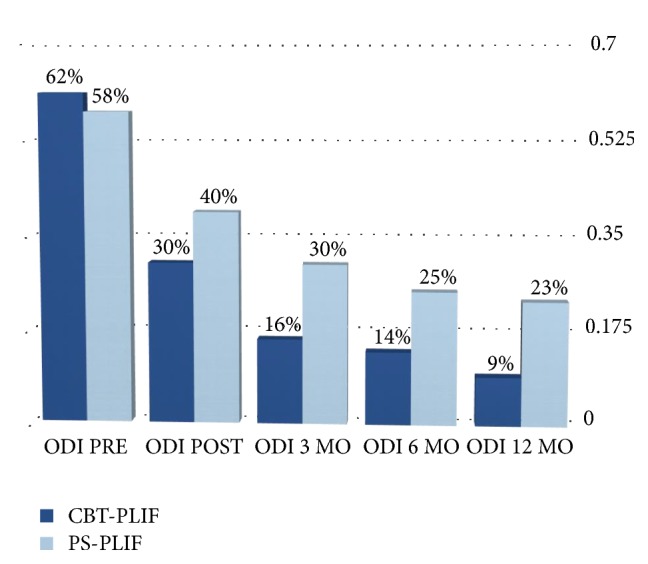
ODI score on admission and at the discharge.

**Figure 4:**
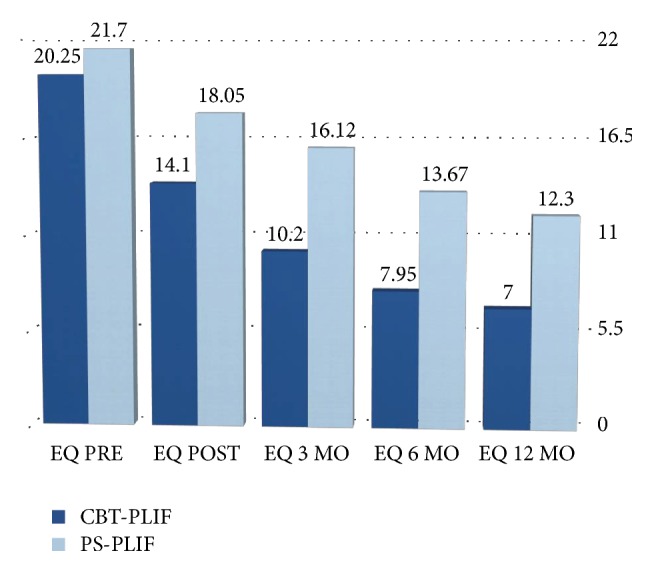
EQ scores on admission and at the discharge.

In addition the mean ODI and EQ scores improved significantly at the discharge in both groups (Figures 3 and 4).

The mean ODI scores in CBT group decreased from 68%  ± 37% to 30%  ± 22% and from 58 ± 15% to 40 ± 8% in the PS group; the mean EQ scores in CBT group decreased from 20.25 ± 4.04 to 14.1 ± 4.93 and from 21.7 ± 2.62 to 18.05 ± 1.90 in the PS group. The ODI and EQ-5D-5L scores at the discharge were also found to be significantly different between the two groups (*p* = 0.04 for ODI and *p* = 0.0358 for EQ index). Mean VAS score for low back pain indicated that 1-year postoperative pain levels were significantly than preoperative ones for both groups, with mean scores decreasing from 8.6 (SD 1.19) to 1.95 (SD 1.47) in CBT group and from 8.25 (SD 1.27) to 2.85 (SD 1.31) in PS group with a *p* value < 0.001. The VAS scores at 12 months were also found to be significantly different between the two groups (*p* = 0.0160). In addition the mean ODI and EQ scores improved significantly at 1 year in both groups. The mean ODI scores in CBT group decreased from 68%  ± 37% to 9%  ± 10% and from 58 ± 15% to 23 ± 9% in the PS group; the mean EQ scores in CBT group decreased from 20.25 ± 4.04 to 7 ± 2.99 and from 21.7 ± 2.62 to 12.05 ± 3.38 in the PS group. The ODI and EQ-5D-5L scores at 12 months were also found to be significantly different between the two groups (*p* = 0.0150 for ODI and *p* = 0.0470 for EQ index) (Table 3).

Statistical comparisons between surgical morbidities (Table 4) showed no difference according to operative time and total radiation dose area product (*p* = 0.0993 and *p* = 0.6913, resp.). On the contrary the two groups had different mean blood loss and length of stay. Average blood loss was 276.50 ml (SD 67.99 ml) for the CBT group and 330.50 ml (SD 90.40 ml) for the PS group; thus the difference can be considered as statistically significant (*p* = 0.0392). The difference between the two groups according to mean length of stay was also significantly different with a mean recovery duration of 2.9 ± 1.37 day for the minimally invasive group and 3.8 ± 1.32 day for the open group (*p* = 0.0413).

Facet joint violation was evaluated on postoperative CT scan according to Seo taxonomy [4]. Facet joint violation occurred in 1 of 80 screws (1.25%) in the CBT group while in the other group 7 screws violated the joint (8.75%). In all cases, screws were either in contact with or suspected to has invaded the facet joint (1 point, on Seo classification).

This comparison cannot be formally defined as statistically significant (*p* = 0.0635) but a trend in any case is evident. There was one case of cage subsidence in the MIS group and two in the open group (*p* = 0.3846). Thus this comparison has to be considered as not significant.

No screw pullout occurred in both groups. There were 3 cases of screw malpositioning in both groups with no neurological complication and one late superficial wound infection that did not require hardware removal and was treated with antibiotic therapy with success. No vascular injury or any other complication has been reported.

We tried to find out whether a correlation between muscular damage and clinical outcomes scores was evident using the measurement of dependence through Pearson's correlation coefficient. For what concerns the relationship between the T2 ratio change in both group and VAS scores we found Pearson's index of 0.75; the correlation was still present with ODI and EQ scores (Pearson's index 0.68 and 0.59, resp.). Furthermore we analyzed the relationship between outcome scores and MF-CSA change in both groups, then founding Pearson's index of 0.78 for VAS score, 0.64 for ODI, and 0.69 for EQ-5Q-5L.

## 4. Discussion

Divergent, cortical trajectory pedicle screws have been proposed as an alternative to standard, convergent pedicle-screw constructs [5–7].

There were several principle findings of the present study. Similar fusion rates were observed in both groups at 12-month follow-up. Clinically, both CBT-PLIF and PS-PLIF provided significant improvement in pain relief and functional status; moreover CBT-PLIF has been proved to be superior to PS-PLIF according to short and long term clinical outcome scores (VAS, ODI, and EQ at the discharge and at 12 months after surgery). This is probably related to the muscular sparing obtained by this new technique and is thus demonstrated by the significant difference in MF-CSA and T2 ratio between the two groups. In addition CBT technique resulted in lower surgical morbidity measured by blood loss, recovery duration, incision length, and superior facet joint violation with subsequent postoperative radiculitis. Even more the overall complication rates (hardware failure, neurological deficits, infections, and vascular injuries) were found to be similar between the two groups.

In our study there were no statistically significant differences among all indications given in the two groups: we enrolled only patients who underwent monosegmental fusion; this was chosen because we had no previous experience on this technique and we assumed that it was safer to begin with one-level fusions.

Clinically, there was a statistically significant difference between the two groups in short and long term improvement in pain intensity, ODI, and EQ scores. CBT-PLIF technique provided significantly better clinical scores compared to PS technique which was probably due by smaller skin incision and less muscle dissection needed to gain the screws' entry point. A shorter skin incision with a reduced use of narcotics, less blood loss, and minimal muscle dissection are all factors contributing to a reduced length of stay and consequently reduced costs for the hospital. Better clinical scores at the discharge in the CBT groups seem to be explained by the reduced muscle dissection; however, it is unclear why this significant difference is still present one year after surgery. This might be probably due to the increased loss of fatty-free functional muscle content that is still evident 1 year after surgery and it is higher in PS group.

The quantification of muscle change following these two different surgical approaches was determined by measuring the change in total muscle area pre- and postoperatively. The examination of functional muscle area by exclusion of fatty and scar tissue is important, since atrophy of the muscle tissue can occur without reducing the cross-sectional area of the muscle within its fascial boundary. Damaged muscle can atrophy and be replaced with fat and fibrous tissue, which results in the reduced functional capacity of the muscle. Therefore, in this study we compared the area of lean muscle tissue between the two surgical approaches and showed that there is a significant change in muscle area as a result of the approach used. In contrast to the CBT approach, at a mean follow-up of 12 months after surgery, there was a reduction in the cross-sectional area of the paraspinal muscles at the surgical site for the open technique. Anatomic damage to the paraspinal muscles has been associated with the occurrence of back muscle dysfunction after surgery [8, 9]. Therefore minimizing the iatrogenic injury to the muscles is significant.

One of the most important concerns for pedicle screw is the risk of superior articular facet violation during screw positioning [10, 11]. Because of the entry point of the being near the pars articularis, which is far from the superior facet joint, the risk of superior facet violation is much lower than in traditional technique. The present study also showed that the facet joint violation occurred in 8.75% in open group and 1.25% in CBT.

Other advantages include a surgical time at least equal to traditional pedicle screw technique, estimated at 90 minutes for a monosegmental fixation with reduced blood loss (<100 cc). Like the traditional lateral-to-medial trajectory, fluoroscopy guidance is needed during surgery. As reported by Rampersaud et al. [12] fluoroscopy-assisted 36 pedicle screw implantation is associated with a 10 to 12 times greater radiation exposure to the spinal surgeon than in nonspinal orthopedic procedures. Although the use of fluoroscopy during the mediolateral approach for pedicle screw placement is equivalent to the traditional technique, as also demonstrated by our study, the dose of radiation to the surgeon is very low when compared to the percutaneous approach, in which a constant use of image guidance is required during surgery [13].

The last advantage of the mediolateral trajectory technique is represented by the differences in the acute hospitalization charges when compared to traditional pedicle screw fixation. In our experience, length of stay (LOS) averaged 3 days.

Nevertheless, the CBT PLIF technique is limited to levels from L1 or L2 (depending on the position of the conus medullaris) down to S1. Previous surgery including extensive laminectomy and/or facetectomy may increase the difficulty or even preclude this technique; in patients with partially or fully destructed articular joints, landmarks for CBT screw insertion may no longer be available. The novel isthmus-guided CBT approach by Iwatsuki et al. may help solve this problem [14, 15].

A radiological preoperative assessment of the lumbar spine with a meticulous planning of entry points for the CBT screws is essential. Guidance systems such as intraoperative fluoroscopy, navigation, or neuromonitoring can be considered by the surgeon depending on the level of expertise and preference.

The present study has some limitations. First, we did not conduct the study with a sufficient sample size because it was initially designed to demonstrate the noninferiority of the new technique versus the “traditional” one so we decided to enroll only patients with at least 12 months of follow-up period. This might be considered a selection bias. Finally, this study was not blinded to the physicians involved in the evaluation of the radiological images and other health-care providers because of the nature of the study type, which may produce some performance bias.

Despite these limitations, the present study has a unique key strength: this is the first clinical European study and the second worldwide with a prospective comparative design to evaluate CBT outcomes in PLIF with a focus on how these outcomes can correlate with the reduced muscular damage.

## 5. Conclusion

The present study was, so far, the first comparative study involving CBT technique in Europe and has been conducted to evaluate the efficacy of CBT screws in PLIF comparing this technique to the traditional pedicle screw technique one year after surgery through a prospective comparative design. There was no significant difference in fusion rate between the two techniques which was the primary endpoint. In addition, a significant difference in muscular damage was found according to the MRI evaluation. Clinical outcomes, based on pain intensity, ODI status, and EQ score, were found also statistically different, even one year after surgery. Moreover, CBT screws in PLIF resulted in less facet joint violation and surgical morbidity than PS. Secondarily, this study also demonstrated a correlation between patients' muscle damage and their clinical picture as already discussed in the previous section. On the basis of these outstanding results, we suggest that CBT screws would provide at least similar outcomes compared to PS in PLIF at one year after surgery, and, thus, CBT is a reasonable alternative to PS in PLIF. Additional studies should be performed with larger sample sizes, extended follow-up periods, and prospective randomized designs to better understand clinical and radiological outcomes of these two techniques.

## Figures and Tables

**Table 1:** Demographic characteristics of the groups.

Demographics	CBT-PLIF	PS-PLIF	*p* value
Age (years)	45,75 (SD 9,63)	54 (SD 12,01)	0,0216
Gender	12/8	9/11	0,1612
Body mass index	27,32 (SD 4,85)	28,82 (4,58)	0,3210
DEXA *T*-score	1,13 (SD 1,20)	1,115 (SD 1,13)	0,9677
Smoke	8/12	12/8	0,1151
Symptoms duration (months)	27 mo (SD 28)	16 mo (SD 9)	0,0938
Cage material (PEEK/tantalum)	4/16	3/17	1,0000
Surgical level (L3L4/L4L5/L5S1)	2/6/12	1/11/8	
Pathology			
*LSS w foraminal stenosis*	1	4	
*Isthmic spondylolisthesis*	/	2	
*Degenerative spondylolisthesis*	1	4	
*Recurrent disc herniation/discopathy*	18	10	

**Table 2:** Radiological outcomes.

Radiological outcomes	CBT-PLIF	PS-PLIF	*p* value
Fusion (12 mo FU)	18/2	17/3	0,3292
Superior facet joint violation	1,25%	8,75%	0,0635
Subsidence (12 mo)	19/1	18/2	0,3846
Pull out (% at 12 mo)	0%	0%	1,0000
MF-CSA % change at 12 mo	16,76% (SD 14,17%)	24,59% (SD 9,68%)	0,0483
T2 ratio % change at 12 mo	9,35% (SD 12,71%)	23,96% (SD 15,99%)	0,0028

**Table 3:** Comparison between clinical outcomes.

Clinical scores	CBT-PLIF	PS-PLIF	*p* value
VAS pre-Vas post (mean)	3,90 (SD 2,25)	2,70 (SD 1,34)	0,0471
ODI pre-ODI post (mean)	31% (SD 25%)	18% (SD 11%)	0,04
EQ pre-EQ post (mean)	6,15 (SD 4,85)	3,65 (SD 1,69)	0,358
VAS pre-Vas 12 mo (mean)	6,65 (SD 1,84)	5,4 (SD 1,23)	0,0160
ODI pre-ODI 12 mo (mean)	52% (SD 26%)	34% (SD 16%)	0,0150
EQ pre-EQ 12 mo (mean)	12,85 (SD 6,08)	9,65 (SD 3,41)	0,0470

**Table 4:** Surgical morbidity.

Surgical morbidity	CBT-PLIF	PS-PLIF	*p* value
Radiation DAP (mGy/cm2)	1960,15 (SD 443,64)	2024,15 (SD 561,24)	0,6913
Surgical time (minutes)	157,45 (SD 21,74)	169,65 (SD 23,87)	0,0993
Length of stay (days)	2,9 (SD 1,37)	3,8 (SD 1,32)	0,0413
Blood loss (cc)	276,5 (SD 67,92)	330,5 (SD 90,41)	0,0392

## Abbreviations

CBT: Cortical bone trajectory
PEEK: Polyetheretherketone
PLIF: Posterior lumbar interbody fusion surgery
PS: Pedicle screws
BMI: Body mass index
*T* score: Bone minerality index
MF-CSA: Multifidus cross-sectional area
ODI: Oswestry Disability Index
EQ scores: Euroquality-5D score
VAS: Visual Analog Scale
TLIF: Transforaminal lumbar interbody fusion
LOS: Length of stay.
